# Gut microbiota and polyendocrine metabolic ovarian syndrome: an integrated gut–metabolism–endocrine–ovary axis

**DOI:** 10.3389/ebm.2026.11058

**Published:** 2026-07-10

**Authors:** Shengyue Jin, Menglei Zhu, Yiyang Lu, Jia Zhao, Jiayi Zhu, Xiaohong Fang

**Affiliations:** Department of TCM Gynecology, Hangzhou TCM Hospital of Zhejiang Chinese Medical University (Hangzhou Hospital of Traditional Chinese Medicine), Hangzhou, Zhejiang, China

**Keywords:** bile acids, gut microbiota, hyperandrogenism, insulin resistance, microbiota-derived metabolites, polycystic ovary syndrome, polyendocrine metabolic ovarian syndrome, short-chain fatty acids

## Abstract

Polyendocrine metabolic ovarian syndrome (PMOS), previously known as polycystic ovary syndrome (PCOS), is a common endocrine and metabolic disorder in reproductive-age women, characterized by marked clinical and biological heterogeneity. Accumulating evidence suggests that gut microbiota dysbiosis is associated with metabolic disturbances, hormonal imbalance, and ovarian dysfunction in PMOS. However, the pathways linking gut microbiota alterations to PMOS pathogenesis remain incompletely understood, and most evidence remains associative. This review aims to summarize current evidence regarding interactions between gut microbiota and PMOS, clarify the roles of key microbiota-derived metabolites, and evaluate the potential and limitations of gut microbiota–targeted interventions. A major novelty is the proposal of an integrated gut–metabolism–endocrine–ovary axis incorporating phenotypic heterogeneity, methodological variability, and evidence grading across clinical and preclinical studies. A narrative review with a systematic literature search was conducted. PubMed, Web of Science, Embase, and CNKI were searched from inception to March 2026 using terms related to PMOS, gut microbiota, microbial metabolites, and microbiota-targeted interventions. Eligible studies included human observational or interventional studies, animal experiments exploring microbiota–PMOS mechanisms, and peer-reviewed full-text articles in English or Chinese. Case reports, letters, conference abstracts, non-English publications, and irrelevant studies were excluded. Duplicate records were removed. Two authors independently screened records and resolved disagreements by consensus. No meta-analysis was performed, and clinical registration was not applicable. Gut microbiota dysbiosis may contribute to PMOS through chronic low-grade inflammation, insulin resistance, and hyperandrogenism. Microbiota-derived metabolites link intestinal dysbiosis with metabolic and endocrine dysfunction. Bile acids and short-chain fatty acids exert regulatory effects, whereas amino acid disorders and LPS-mediated endotoxemia amplify metabolic and inflammatory abnormalities. Considerable heterogeneity exists across studies regarding obesity, insulin resistance, hyperandrogenism, diet, ethnicity, region, and methodology. Microbiota-targeted interventions show potential, although evidence quality varies and most findings remain associative. Gut microbiota dysbiosis is a critical regulatory node within the integrated gut–metabolism–endocrine–ovary axis in PMOS. This review highlights phenotypic stratification, evidence hierarchy, and clinical translation potential. Although microbiota-targeted strategies may serve as adjunctive therapies, their causal roles and long-term efficacy require confirmation in well-designed longitudinal and randomized controlled trials.

## Impact statement

Polycystic ovary syndrome (PCOS) is a complex disorder with diverse metabolic and reproductive features, yet its underlying mechanisms remain incompletely understood. Growing evidence links changes in gut microorganisms to insulin resistance, hormone imbalance, and ovarian dysfunction in PCOS, but findings have been fragmented. This review integrates current knowledge into a unified “gut-metabolism-endocrine-ovary” framework, highlighting how gut-derived molecules may connect intestinal imbalance with metabolic and reproductive disturbances. We distinguish between microbial factors that help maintain balance and those that may amplify inflammation and metabolic disruption, offering a clearer understanding of disease heterogeneity. By synthesizing mechanistic insights and evaluating emerging microbiota-based interventions, this work advances the field beyond associative observations toward a more structured, system-level perspective. These insights may guide future longitudinal and interventional studies and support the development of more precise, phenotype-oriented strategies for PCOS management.

## Introduction

Polyendocrine metabolic ovarian syndrome(PMOS) previously known as polycystic ovary syndrome (PCOS),is a prevalent endocrine and metabolic disorder affecting women of reproductive age, manifesting as menstrual irregularity, alopecia, acne, hirsutism, and infertility [1, 2]. It is defined by hyperandrogenism, ovulatory dysfunction, and polycystic ovarian morphology, and is frequently accompanied by insulin resistance (IR), obesity, and chronic low-grade inflammation [3]. Hyperandrogenemia and insulin resistance are core pathogenic drivers that disrupt endocrine function and promote adiposity, exacerbating obesity-related complications. These disturbances not only directly impair endocrine homeostasis but also aggravate fat accumulation, thereby exacerbating the progression of obesity [4]. Globally, PMOS affects 10%–15% of reproductive-age women and is diagnosed using the Rotterdam criteria [5]. As the most widely accepted diagnostic standard, the Rotterdam criteria specify that PMOS can be diagnosed when two of the following three conditions are present, after excluding other etiologies of androgen excess: oligo-ovulation or anovulation; clinical and biochemical hyperandrogenemia; and polycystic ovarian morphology(PCOM) [6]. Long-term complications include cardiovascular disease and type 2 diabetes, imposing a considerable health burden [7]. Despite extensive research, the pathophysiological basis of PMOS remains incompletely understood due to high phenotypic heterogeneity.

In recent years, many reviews have addressed gut microbiota in PMOS. However, most prior reviews remain descriptive, lack an integrated mechanistic framework, and fail to systematically evaluate clinical evidence quality, phenotypic stratification, and causal uncertainty [8]. The present review fills these gaps by establishing a unified gut–metabolism–endocrine–ovary axis and by critically appraising evidence with explicit attention to clinical translatability and phenotypic specificity. This represents a key improvement over existing reviews.

Recent studies have provided growing evidence suggesting a significant association between the pathogenesis of PMOS and dysregulations in the composition and function of the gut microbiota [9]. The gut microbiota, a complex community of microorganisms residing within the gastrointestinal tract, plays a crucial role in maintaining metabolic, immune, and endocrine homeostasis [10]. The gut microbiota of PMOS patients may be associated with the development and occurrence of hyperandrogenism, IR, chronic inflammation, and metabolic syndrome, and may influence the clinical manifestations of PMOS via bile acids (BAs), short-chain fatty acids (SCFAs), amino acids (AAs) and lipopolysaccharides(LPS) [11, 12]. Alterations in the gut microbiome may influence the reproductive endocrine system, potentially affecting follicular development and ovulatory function, and thereby impacting reproductive outcomes in PMOS patients [13].

Current therapeutic strategies for PMOS primarily focus on symptomatic management, including lifestyle interventions and hormonal therapies to regulate menstrual cycles [14]. However, these treatments fail to address the underlying pathophysiological mechanisms of PMOS, such as insulin resistance, metabolic dysfunction, and gut microbiota dysbiosis. Based on the available evidence, we propose an integrated “gut–metabolism–endocrine–ovary axis” framework for PMOS. Gut microbiota dysbiosis and impaired intestinal barrier function may alter the profile of microbiota-derived metabolites, thereby potentially driving chronic low-grade inflammation and insulin resistance. These alterations may subsequently promote hyperandrogenic states and exacerbate follicular development impairment and ovulatory dysfunction. Conversely, hyperandrogenism, insulin resistance, and obesity may reciprocally reshape gut microbial ecology, forming a self-reinforcing pathological cycle.

**FIGURE 1:**
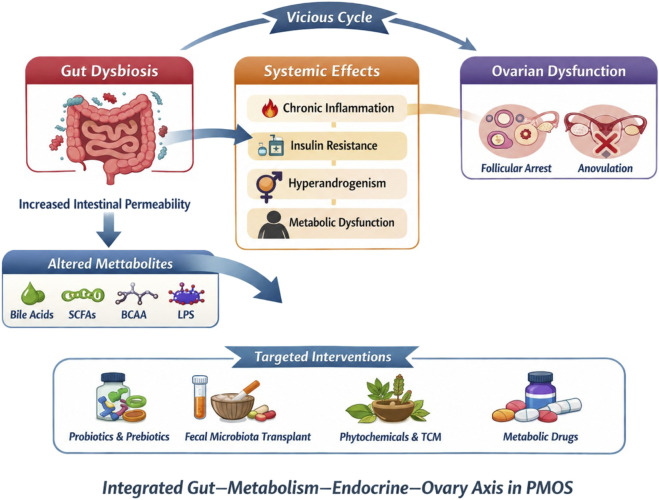
Schematic overview of the integrated gut–metabolism–endocrine–ovary axis in PMOS.

Elucidating key nodes and causal relationships within gut microbiota–metabolite–host signaling pathways across different PMOS phenotypes may not only help explain the marked clinical heterogeneity of the syndrome but also provide critical insights for development of microbiota-based, stratified intervention strategies. Accordingly, this review systematically summarizes recent advances in understanding PMOS–gut microbiota interactions, with particular focus on driving factors of microbial dysbiosis, mechanistic networks mediated by key microbiota-derived metabolites, and progress in microbiota-targeted therapeutic approaches. In addition, current research limitations and future directions are discussed to offer new perspectives and potential clinical implications for improving metabolic and reproductive outcomes in patients with PMOS. The key microbiota-derived metabolites and related evidence are summarized in Table 1, and the proposed integrated gut–metabolism–endocrine–ovary axis is illustrated in Figure 1.

**TABLE 1:** Key microbiota-derived metabolites and related evidence in the integrated gut–metabolism–endocrine–ovary axis in PMOS.

Metabolite	Core mechanisms	Clinical evidence in humans	Evidence level	Representative interventions
Bile acids	Regulate FXR/TGR5 signaling; modulate insulin sensitivity; associated with hyperandrogenism and ovarian dysfunction	Altered profiles in PMOS; limited clinical intervention data	Limited clinical evidence	Bile acid modulation, UDCA, dietary intervention
Short-chain fatty acids	Enhance intestinal barrier; reduce inflammation; improve insulin sensitivity; regulate immune balance	Reduced butyrate levels in PMOS; few clinical trials	Preclinical + small human studies	Probiotics, prebiotics, dietary fiber
Branched-chain amino acids	Promote IR; activate mTOR signaling; correlate with hyperandrogenism and metabolic severity	Elevated in PMOS; linked to IR and obesity	Moderate clinical association	Dietary control, microbiota modulation
Lipopolysaccharide (LPS)	Induces metabolic endotoxemia; activates TLR4/NF-κB; aggravates inflammation and IR	Elevated circulating LPS; strong association only	Strong association; no causal proof	Gut barrier protectors, anti-inflammatory agents

## Literature search strategy and review methods

This article was designed as a narrative review with a systematic literature search, aiming to summarize current evidence on the association between gut microbiota and PMOS, previously known as PCOS, based on peer-reviewed literature. Literature retrieval was carried out across four electronic databases: PubMed, Embase, Web of Science, and China National Knowledge Infrastructure (CNKI). The search period covered all available publications from the inception of each database up to March 2026. The search strategy was constructed using combinations of key terms related to PMOS, gut microbiota, microbial metabolites, and microbiota-targeted interventions.

Studies were considered eligible if they focused on the composition, function, or metabolic profiles of gut microbiota in patients with PMOS or in corresponding animal models, investigated mechanistic pathways connecting gut microbiota to metabolic regulation, endocrine homeostasis, inflammatory activation, and ovarian function, or assessed the therapeutic effects of interventions targeting the gut microbiota, including probiotics, prebiotics, fecal microbiota transplantation, lifestyle modification, natural compounds, and pharmacological agents. Only full-text peer-reviewed articles written in English or Chinese were included.

Studies were excluded if they were case reports, editorials, letters, expert comments, conference abstracts, or publications with no clear relevance to gut microbiota or the pathogenesis of PMOS. Duplicate records and articles with incomplete or unreliable data were also excluded from the final analysis.

Two independent reviewers performed initial screening of all retrieved records based on titles and abstracts. Records deemed potentially eligible were further evaluated through full-text assessment. Any discrepancies during the screening process were resolved through discussion with a third reviewer until a unanimous consensus was achieved. Relevant information was extracted systematically, including study design, study population or animal model, characteristics of gut microbiota, key microbial metabolites, types of intervention measures, and major outcome indicators.

## The relationship between gut microbiota and PMOS

The gut microbiota, commonly described as the host’s “second genome,” is primarily composed of the phyla Bacteroidetes and Firmicutes, with additional contributions from Proteobacteria, Verrucomicrobia, Actinobacteria, Clostridia, and Cyanobacteria [15]. This microbiota plays a critical role in digestion, nutrient uptake, energy harvest, vitamin synthesis, inflammatory modulation and host immune status [16]. Furthermore, the gut microbiota has been recognized as an endocrine-like organ that contributes to the regulation of host metabolic and hormonal homeostasis. The gut microbiota influences the reproductive endocrine system through interactions with sex hormones and metabolic regulators, including estrogen, androgens, and insulin [17]. A marker of good gut health is high bacterial diversity and functional diversity, along with an intact intestinal barrier [18]. A healthy gut harbors greater abundance of Bifidobacterium and *Lactobacillus* species, which enhance the intestinal barrier, modulate immune responses, and antagonize pathogens [19]. In contrast, dysbiosis refers to an imbalance in the composition and function of the gut microbiota, characterized by a reduction in beneficial microbes and an overgrowth of potentially harmful ones [15].

Accumulating evidence indicates that PMOS is associated with decreased microbiota diversity, reduced beneficial bacteria, and increased pro-inflammatory species [20, 21]. A recent review further summarized that altered gut microbial composition in PCOS is closely associated with metabolic abnormalities, including insulin resistance, obesity-related metabolic dysfunction, and chronic inflammation [22]. This dysbiosis may contribute to the development of hallmark features of PMOS, such as metabolic dysfunction, hormonal imbalance, and chronic low-grade inflammation [23, 24]. Under physiological conditions, the integrity of the intestinal barrier is essential for nutrient absorption, pathogen defense, and maintenance of gut homeostasis. Intestinal epithelial cells preserve barrier integrity through tight junction proteins [25]. Moreover, gut microbes enhance intestinal barrier function by upregulating tight junction protein expression, reducing circulating endotoxins, and stabilizing gut immunity [26]. However, dysbiosis can exacerbate inflammatory responses by triggering the release of inflammatory cytokines, compromising intestinal barrier integrity, and facilitating the translocation of lipopolysaccharides (LPS) into the systemic circulation [27]. In conclusion, the gut microbiota may influence the pathogenesis of PMOS through mechanisms such as hyperandrogenism, IR, and chronic inflammation, thereby affecting follicular development, sex hormone levels, and metabolic state.

### Impact of PMOS on gut microbiota

#### Effect of sex hormones on gut microbiota

Sex hormones are known to regulate gut microbiota composition in a sex-dependent manner. Elevated androgen levels have been associated with reduced gut microbiota diversity, characterized by an increased Firmicutes-to-Bacteroidetes ratio, enrichment of genera such as *Clostridium*, Dorea, and Bilophila, and a marked reduction in beneficial bacteria, including *Lactobacillus* species [28]. Studies using dihydrotestosterone-induced PMOS rat models have shown increased body weight, fat deposition, and decreased intestinal flora diversity, indicating that the gut microbiota is related to high androgen levels [29]. Sex hormone status significantly alters intestinal flora structure and metabolite profiles, including short-chain fatty acids and indole derivatives, suggesting that hyperandrogenism in PMOS may disrupt flora homeostasis and alter metabolic output, leading to metabolic disorders [30].

Hyperandrogenism in PMOS patients is mainly driven by enhanced ovarian steroidogenesis, increased adrenal 11-oxidized androgen secretion, and IR, which further promote androgen production and reduce sex hormone-binding globulin levels, exacerbating androgen excess [31]. The intestinal flora of women with PMOS shows increased abundance of Catenibacterium and Kandleria genera, and bacterial diversity indices are positively correlated with serum androgen concentrations and negatively correlated with estradiol levels [32]. Estrogen and progesterone preserve bacterial diversity and metabolic balance, while PMOS patients frequently exhibit elevated estrogen and insufficient progesterone. The hormonal imbalance, together with high androgen levels, alters gut microbiota composition, increase proinflammatory bacteria, reduces probiotics, and worsens metabolic disorders [33].

Collectively, the hormonal dysregulation observed in patients with PMOS may profoundly alter metabolic pathways, disrupt intestinal barrier integrity, and modulate immune responses, thereby potentially influencing the composition of gut metabolites and microbiota. However, these observations are largely associative and predominantly derived from cross-sectional studies and animal models. Causal evidence from large prospective cohorts or randomized trials is lacking. Furthermore, microbial signatures vary considerably according to obesity status, androgen severity, dietary intake, ethnicity, and geographic region, leading to inconsistent findings across studies.

#### Effect of IR on gut microbiota

IR is one of the most prevalent metabolic abnormalities in patients with PMOS, affecting approximately 50–70% of this population to varying degrees [20]. IR exacerbates disturbances in glucose and lipid metabolism and contributes to hyperandrogenemia [34]. The presence of IR may significantly influence the diversity of gut microbiota and the abundance of specific bacterial taxa in patients with PMOS. Among women with IR, the relative abundance of *Bacteroidaceae* increased, whereas *Prevotellaceae* decreases compared with PMOS women without IR [35]. Increased Bacteroidaceae is positively correlated with inflammation and insulin resistance severity [36].

Experimental studies show that *Enterococcus* and Rothia are markedly increased in PMOS patients with IR and positively linked with waist circumference, free fatty acids, and HOMA-IR index, whereas Prevotella is dramatically diminished. IR may be associated with impaired GLP-1 signaling, aggravated lipid metabolic dysfunction, and enhanced inflammatory responses [37]. IR also affects functional alterations within the gut microbiota; patients with insulin resistance exhibit elevated LPS biosynthesis pathways and heightened inflammatory cytokines, suggesting increased inflammatory risk [36]. IR-induced gut dysbiosis potentiates branched-chain amino acid production and impairs their clearance, contributing to their accumulation [11]. Moreover, IR and compensatory hyperinsulinemia can lead to hyperandrogenism, further exacerbating intestinal flora imbalance and metabolic disorders [38].

Therefore, IR may aggravate intestinal flora imbalance in PMOS patients, and the intestinal flora may represent a potential metabolic regulatory target for PMOS-related IR [39]. Nonetheless, causal relationships remain unconfirmed. Most studies are observational and limited by small sample sizes, cross-sectional design, and insufficient adjustment for confounders including diet, medication, and body composition. Heterogeneity in insulin resistance severity and PMOS phenotype further reduces comparability across investigations.

#### Effect of obesity on gut microbiota

There is a bidirectional relationship between obesity and PMOS, with approximately 50%–60% of women with PMOS being overweight or obese. Mendelian randomization studies have identified body mass index(BMI) as a potential causal factor in the development of PMOS [40]. Notably, compared with non-obese PMOS patients, obese PMOS patients exhibit more pronounced alterations in the composition and diversity of their gut microbiota [6]. Visceral obesity substantially modifies intestinal microbiota composition, leading to increased *Prevotella*, *Dialister*, and *Phascolarctobacterium*, alongside alterations in lipid metabolism-related functional pathways. These alterations may exacerbate fat accumulation and insulin resistance, suggesting that the gut microbiota may act as an important mediator linking obesity-related PMOS and metabolic disorders [41].

*Prevotella* and *Bacteroides* are elevated in obese PMOS whereas beneficial microbes including *Akkermansia* and *Ruminococcaceae are* reduced [42]. Obese PMOS patients show higher concentrations of LBP, GLP-2, succinate, and zonulin family peptides, implying heightened intestinal permeability and compromised barrier function, which contribute to endotoxemia and systemic inflammation. Obesity exacerbates the detrimental impacts of intestinal microbiota and barrier impairment in PMOS, hastening the onset of metabolic diseases [43]. Disruptions in gut flora might result in diminished amounts of SCFAs, a microbiome metabolite, and lowered leptin concentrations, thereby hastening the advancement of obesity. Elevated Gram-negative bacteria increase LPS levels, precipitating metabolic endotoxemia [44]. Reduced gut microbiota diversity associated with obesity may reduce resource utilization efficiency and increase vulnerability to dysbiosis [45].

Consequently, obesity appears to be a key factor exacerbating gut microbiota dysbiosis in patients with PMOS. It is important to emphasize that these relationships are associative, not definitively causal. Obese and non-obese PMOS subgroups exhibit distinct microbial profiles, and differences in dietary behavior, physical activity, and socioeconomic background further confound interpretations [46]. Methodological differences in sequencing and bioinformatics also contribute to inconsistent results.

### Impact of gut microbiota on PMOS

#### Effects of bile acids in PMOS

Bile acids (BAs) represent an important class of microbiota-derived metabolites and signaling molecules that influence gut microbiota composition and host metabolism in individuals with PMOS [47]. BAs regulate metabolic homeostasis primarily through activation of bile acid receptors including TGR5 and FXR. Alterations in BA levels may result in metabolic disorders frequently observed in PMOS [16], such as elevated cholesterol, triglycerides, and low-density lipoprotein [48]. Intestinal microbiota can activate Fibroblast Growth Factor 15/19 signaling and suppress cytochrome P450 7A1(CYP7A1) and 8B1(CYP8B1) enzymes implicated in bile acid synthesis, thus modulating triglyceride(TG), LDL, and glucose homeostasis [49].

Animal studies show that ursodeoxycholic acid(UDCA) administration enhances ovarian morphology and decreases testosterone and insulin levels [50]. Clinical studies show that elevated *Bacteroides*, glycodeoxycholic acid, and tauroursodeoxycholic acid correlate with insulin resistance and ovarian dysfunction and exacerbate metabolic and reproductive impairment [51]. Bile acids enhance IL-22 secretion by activating the GATA3 pathway in ILC3s, implying that IL-22 may ameliorate insulin resistance, ovarian function, and estrous cycle abnormalities. Primary bile acid proportions are positively correlated with total testosterone and free androgen index, indicating a link with hyperandrogenism [52]. Glycochenodeoxycholic acid is positively correlated with FSH and LH, and chenodeoxycholic acid-3-β-d-glucuronide increases with antral follicle count, implying roles in follicular development and ovulatory disorders [53].

Based on available experimental and clinical evidence, bile acids may function as a signaling hub linking the gut microbiota with host metabolic and endocrine regulation, exerting modulatory effects on the development of insulin resistance and hyperandrogenic phenotypes in PMOS. However, most clinical evidence remains correlational. Variability in bile acid profiling methods, patient ethnicity, body weight, and PMOS subtype limits generalizability. Causal validation in humans is still limited.

#### Effects of short-chain fatty acids in PMOS

SCFAs are metabolites generated during intestinal microbial fermentation of dietary fiber, mainly including acetic acid(AA), propionic acid(PA), butyric acid(BA), and valeric acid(VA). These compounds are essential for maintaining microbial homeostasis and intestinal mucosal barrier integrity [54]. Circulating butyrate levels are significantly reduced in obese patients with PMOS and closely associated with gut microbial dysbiosis [55]. BA impedes METTL3-mediated FOSL2 m6A methylation, obstructing NLRP3 inflammasome activation and pro-inflammatory cytokines, implying that reduced short-chain fatty acid levels may result in intestinal microbiota dysbiosis and mucosal barrier impairment, exacerbating chronic inflammation [56].

SCFAs can enhance IL-22 secretion via GPR41 signaling and HDAC inhibition, fortifying the intestinal mucosal barrier and mitigating inflammation [57]. They limit pro-inflammatory response in dendritic cells and macrophages by activating GPR41/43 and GPR109A receptors, decreasing IL-6 and IL-12, and facilitating Treg differentiation and IL-10 secretion to sustain immunological homeostasis [58]. Given that patients with PMOS display Treg/Th17 imbalance and abnormal HSP70 expression [59], SCFA-mediated enhancement of IL-22 synthesis and restoration of Treg function may represent a viable strategy to ameliorate immune dysregulation and chronic inflammation in PMOS. The development and reinstatement of Treg function could serve as a possible method for ameliorating immune dysregulation and chronic inflammation associated with PMOS. Furthermore, SCFAs participate in metabolic regulation by enhancing glucose-stimulated insulin secretion and improving insulin sensitivity. Specifically, SCFAs stimulate incretin production via FFAR2/3, thereby augmenting glucose-dependent insulin secretion. SCFA supplementation has been shown to improve fasting insulin levels and HOMA-IR [60]. Research indicates that SCFAs enhance the secretion of multiple gut hormones, including GLP-1, PYY, and 5-HT, through activating FFA2/3 receptors. This effect partially corrects gut hormone secretion disorders in patients with PMOS, thereby sustaining insulin homeostasis and augmenting insulin sensitivity [61].

In summary, SCFAs maintain intestinal barrier integrity and immune homeostasis, thereby attenuating chronic low-grade inflammation and enhancing insulin sensitivity, which may slow the progression of PMOS. However, current evidence is predominantly derived from preclinical studies, whereas clinical data in humans remain limited and inconsistent. Optimal concentrations, administration routes, and long-term safety profiles of SCFA interventions in humans have not been established. Moreover, most existing evidence indicates associations rather than definitive causal relationships.

#### Effects of amino acids in PMOS

AAs are nitrogen-containing organic compounds that serve as the building blocks of proteins and participate in the tricarboxylic acid(TCA) cycle and mitochondrial oxidative phosphorylation [62], regulating lipid and glucose metabolism and influencing insulin signaling. Women with PMOS exhibit significant AA metabolic dysfunction, with specific AAs intimately associated with increased risks of obesity, insulin resistance, and metabolic syndrome [63]. BCAAs, including valine, isoleucine, and leucine, are strongly correlated with metabolic abnormalities in PMOS [64].

The branched-chain amino acid metabolite 3-HIB enhances transendothelial fatty acid transport and uptake, stimulating lipid deposition in skeletal muscle and contributing to insulin resistance [65]. Excess BCAAs and abnormal breakdown trigger the mTORC1/S6K1 pathway and disrupt mitochondrial function and insulin signaling [66, 67]. Elevated plasma BCAA levels are positively correlated with androgen levels, antral follicles, and menstrual irregularity. Defects in BCAA metabolism can exacerbate IR, hyperandrogenism, and obesity, with Mg^2+^/Mn^2+^-dependent protein phosphatase 1K(PPM1K) identified as a causative gene [68]. Furthermore, BCAAs may also exacerbate chronic inflammation by stimulating pro-inflammatory gene expression [11].

Current evidence suggests that abnormalities in amino acid metabolism may contribute to the development of metabolic phenotypes in PMOS; however, compared with bile acids and short-chain fatty acids, their causal role within the gut microbiota–PMOS axis remains less well defined. These alterations are therefore more likely to function as amplifying factors of metabolic dysregulation rather than as primary initiating drivers of the disease process. Furthermore, most human studies are observational and do not exclude confounding by diet, obesity, or insulin resistance. Causal mechanistic data are largely restricted to animal models.

#### Effects of lipopolysaccharide in PMOS

LPS is a major structural component of Gram-negative bacteria and a powerful stimulator of inflammatory responses. Heightened intestinal permeability in PMOS patients facilitates entry of excess LPS into the bloodstream, resulting in endotoxemia that aggravates metabolic disorders and chronic inflammation [69]. High-fat diet increases circulating LPS in mice, and LPS administration induces insulin resistance and obesity [70]. PMOS patients exhibit systemic low-grade inflammation marked by increased peripheral blood LPS and inflammatory markers, which correlate positively with insulin resistance indices.

LPS activates the LPS/TLR4 inflammatory pathway, promote NF-κB activation, and enhances inflammatory responses contributing to insulin resistance [71]. LPS enhances TLR2 expression, stimulates pro-inflammatory mediators including TNF-α, IL-1β, IL-6, and IL-8 in granulosa cells, and initiates oxidative stress [72]. LPS also stimulates the PI3K/AKT/mTOR pathway, facilitating inflammatory molecule production and suppressing autophagy [73]. Fecal metagenomic data suggest that reduced Bacteroidales may elevate pro-inflammatory LPS levels in follicles, correlating with follicular hypoplasia, recruitment difficulties, and elevated AMH levels, indicating an intimate link between gut microbiota and reproductive function [74].

Consequently, LPS-mediated endotoxemia is more likely to function as a key amplifying pathway linking intestinal barrier dysfunction with systemic inflammatory responses, thereby exerting a sustained aggravating effect on metabolic abnormalities and disruption of the ovarian microenvironment in PMOS. While LPS-induced endotoxemia is considered an important amplifier of inflammation and metabolic disorders in PMOS, direct clinical evidence supporting causality remains limited. Most studies are observational or based on animal models, and factors such as intestinal barrier function, microbial source, and individual differences may affect the consistency of results.

Gut microbiota dysbiosis impairs intestinal barrier function and alters the production of key microbial metabolites, including bile acids, short-chain fatty acids, branched-chain amino acids, and lipopolysaccharide. These changes trigger systemic low-grade inflammation and insulin resistance, which further promote hyperandrogenism, follicular developmental arrest, and ovulatory dysfunction. Conversely, hyperandrogenism, insulin resistance, and obesity reciprocally remodel gut microbiota composition, forming a self-reinforcing pathological cycle. Microbiota-targeted interventions may break this cycle and improve PMOS-related phenotypes.

## Gut microbiota–based interventions for PMOS

### Lifestyle interventions

Recent international evidence-based guidelines for PMOS indicate that lifestyle intervention is fundamental to PMOS management [75], with diet and exercise as critical components. Dietary habits alter gut microbiota and significantly influence weight management, insulin sensitivity, and inflammatory markers [76]. Ketogenic diets improve anthropometric and metabolic indices and sex hormone profiles by reducing insulin, suppressing inflammation, and decreasing fat mass [77]. The Mediterranean diet alleviates chronic inflammation, improves insulin sensitivity, and lowers androgen levels, reducing long-term cardiometabolic and reproductive risks [78].

Various exercise modalities including continuous aerobic exercise, high-intensity interval training, and yoga [79–81]improve cardio-metabolic performance, insulin sensitivity, and menstrual regularity. Exercise combined with dietary management improves HOMA-IR, lipid profiles, BMI, waist circumference, and body fat percentage [82]. However, insufficient data prevent direct comparisons between exercise and dietary therapy or between combined interventions and diet alone.

Despite the well-recognized benefits of lifestyle interventions in improving metabolic and reproductive parameters in women with PMOS, it remains difficult to determine whether such beneficial effects are directly mediated by alterations in gut microbiota composition or function, or indirectly attributed to weight reduction, improved energy metabolism, and reduced systemic inflammation. Most existing studies lack strict control over dietary fiber intake, dietary patterns, physical activity intensity, and other confounding factors, and few investigations adopt microbiota-targeted indicators as primary endpoints. These limitations restrict the mechanistic evidence linking lifestyle modifications to gut microbiota regulation in PMOS management.

### Microbiota-based interventions

Gut microbiota–targeted interventions including fecal microbiota transplantation (FMT), probiotics, and prebiotics have gained attention as potential strategies to restore microbial balance and ameliorate metabolic and reproductive dysfunctions in PMOS. FMT transfers healthy donor fecal material to recipients to address dysbiosis-related disorders [83]. Given the consistent association between gut microbial dysbiosis and the core pathophysiological features of PMOS, reconstruction of a healthy gut microbiota community via FMT represents a theoretically feasible intervention for this syndrome.

A previous study using a DHT-induced PMOS mouse model has shown that FMT from healthy mice effectively modified the gut microbiota composition of PMOS mice, aligning it closer to that of healthy controls. Although FMT did not significantly improve overall PMOS-related phenotypic features, it partially reduced fat accumulation and modified the relative abundance of several metabolism-associated bacteria, including *Barnesiella*, *Parabacteroides*, and *Alistipes* [84]. Another study conducted in letrozole-induced PMOS rats enhanced estrous cyclicity, decreased serum androgen concentrations, reinstated normal ovarian morphological structure, and normalized the composition of gut microbiota [85]. Nevertheless, no clinical trials or human observational studies have yet evaluated the safety and efficacy of FMT in women with PMOS, and all available evidence is restricted to preclinical animal models. As a multisystem endocrine and metabolic disorder, PMOS shares overlapping metabolic disturbances with obesity and type 2 diabetes, conditions in which FMT has shown preliminary therapeutic potential [86]. Therefore, FMT remains a promising exploratory strategy for PMOS management, yet its clinical value cannot be confirmed until rigorous human studies are performed.

Similarly, probiotics, defined as “live microorganisms that, when administered in adequate amounts, confer health benefits on the host”, have attracted extensive attention for their potential to ameliorate metabolic and endocrine disorders in PMOS [87]. Accumulating evidence indicates that probiotics improve metabolic profiles and attenuate IR by promoting the production of SCFAs, regulating inflammatory and oxidative stress pathways, and inhibiting the release of proinflammatory cytokines [88]. The probiotic strains with documented beneficial effects in PMOS models mainly belong to the genera *Bifidobacterium*, *Lactobacillus*, *Clostridium*, *Enterococcus*, and other lactic acid bacteria [89]. Research indicates that Bifidobacterium longum subsp. Longum BL21 can mitigate PMOS by enhancing hormonal levels in PMOS mice via the brain-gut-ovary axis, curtailing weight gain, diminishing inflammatory markers, and optimizing microbial composition. However, this study did not fully elucidate the interactive mechanisms among dietary metabolism, gut microbiota, and PMOS pathogenesis [90].

He et al. [91] employed a letrozole-induced PMOS rat model to systematically evaluate the therapeutic effects of eight lactic acid bacterial strains. The results showed that *Lactobacillus* plantarum HL2 and Bifidobacterium longum HB3 significantly ameliorated ovarian pathological alterations, diminished testosterone levels, and partially restored LH, FSH, and their abnormal ratios. Furthermore, these strains modulated gut microbiota associated with sex hormones, including *Akkermansia*, *Prevotella*, and *Staphylococcus*.

Prebiotics are specialized substrates that are selectively utilized by host microorganisms and confer beneficial effects on host health [87]. Indigenous intestinal microorganisms ferment prebiotics to produce bioactive metabolites, among which oligofructose and oligogalactose are the most widely studied for their health-promoting properties [92]. Certain studies indicate that prebiotics can mitigate hyperandrogenism, enhance insulin sensitivity, and lower cardiometabolic risk by fostering the synthesis of SCFAs in the colon, ameliorating inflammatory conditions, and optimizing energy metabolism [93].

Animal studies have shown that inulin supplementation improves glucose tolerance, insulin sensitivity, and ovarian morphology in PMOS models, while reducing serum testosterone, AMH, and LH levels. These effects were accompanied by increased abundance of SCFA-producing bacteria, enhanced acetate, propionate, and butyrate production, improved intestinal barrier function, and suppression of inflammatory mediators such as IL-1β, IL-18, and LBP. A clinical investigation including women with PMOS has shown that supplementation with 10 g of inulin over three consecutive months improved hyperandrogenism, IR, and dyslipidemia, while increasing *Bifidobacterium* abundance and decreasing *Escherichia coli* and *Shigella* levels [27]. A further investigation assessed the impact of resistant dextrin on enhancing metabolic indices, androgen levels, and clinical symptoms, including hirsutism and menstrual cycle abnormalities, in women with PMOS [94].

Based on current evidence from animal models and limited human studies, gut microbiota–targeted interventions—including fecal microbiota transplantation, probiotics, and prebiotics—are more likely to exert adjunctive therapeutic effects in PMOS by improving the metabolic and inflammatory microenvironment, rather than serving as independent or definitive treatment modalities. Although the observed beneficial effects provide indirect support for the causal involvement of gut microbiota in PMOS pathophysiology, the stability, efficacy reproducibility, and clinical generalizability of these interventions remain to be validated through well-designed, large-sample, and long-term randomized controlled trials with strict phenotypic stratification. A recent systematic review and meta-analysis of randomized controlled trials further suggested that microbiota-related interventions may improve several metabolic, hormonal, and inflammatory outcomes, but heterogeneity in intervention type, duration, and outcome definitions remains an important limitation [95].

### Natural plant-derived compounds

Natural plant-derived compounds, including polyphenols and traditional Chinese medicines (TCM) show considerable potential in modulating gut microbiota composition, alleviating inflammation, and improving metabolic and endocrine disturbances in PMOS. These substances promote beneficial bacteria, restore homeostasis, and attenuate systemic inflammation, contributing to improved hormonal balance, insulin sensitivity, and lipid metabolism [96]. Polyphenols are a large class of plant secondary metabolites, mainly including resveratrol, anthocyanins, catechins and other components. Numerous studies indicate that resveratrol enhances insulin sensitivity by activating SIRT1/AMPK and PI3K/Akt signaling pathways [97, 98], modulates PPAR-γ and lipid metabolism-related variables, and mitigates dyslipidemia. Simultaneously, resveratrol can reduce androgen synthesis by inhibiting the activities of CYP17A1 and HMG-CoA reductase [99]. In addition, resveratrol can improve chronic low-grade inflammation by blocking NF-κB and VEGF signaling pathways, and restore normal ovulatory function and follicle morphology [100]. Notably, resveratrol also exerts a prebiotic-like effect, which can improve gut microbiota dysbiosis associated with PMOS, thus indirectly alleviating metabolic disorders. Although the safety of resveratrol *in vivo* is satisfactory, its clinical efficacy as an adjuvant therapy for PMOS still needs to be confirmed by high-quality randomized controlled trials [99].

Anthocyanins have been proved to improve ovarian steroid hormone imbalance in androgen-induced PMOS mice, regulate steroid synthase and antioxidant enzyme levels, and address inflammatory marker abnormalities, demonstrating potential therapeutic effects [101]. Research indicates that catechins in oolong tea can improve ovarian dysfunction and insulin resistance in PMOS mice by inhibiting the p-STAT3 signaling pathway, reduce uterine inflammation and matrix degradation [102]. Green tea is abundant in catechins, and green tea extract can enhance IR and ovarian morphology in PMOS model rats while decreasing testosterone levels [103].

Traditional Chinese medicine has gradually formed a unique therapeutic system for PMOS under long-term clinical practice. This system is guided by the holistic view and treatment based on syndrome differentiation, and has the characteristics of multi-target and multi-pathway regulation. A large number of studies have confirmed that TCM can significantly ameliorate endocrine disorders in PMOS patients by regulating ovarian blood flow dynamics, improving serum hormone levels and normalizing menstrual cycles, so as to increase ovulation rate and clinical pregnancy rate.

In recent years, the regulatory effect of TCM and its components on gut microbiota has been gradually revealed. Studies have shown that Bailing capsule can improve ovarian dysfunction, reduce IR, and enhance sex hormone levels in mice with PMOS. BL modulates the intestinal microbiota and inflammatory response in PMOS model mice. Bailing capsule can improve gut microbiota disturbance and inflammatory response in PMOS mice by regulating LPS-TLR4 inflammatory pathway [104]. Other TCM formulations, including HeQi San, Bu Shen Hua Zhuo formula, Zishen Qingre Lishi Huayu formulation, and Yulin Tong Bu compound, have also been shown to alleviate PMOS-like phenotypes in animal or clinical studies by improving ovarian function, enhancing insulin sensitivity, increasing gut microbiota diversity, and enriching beneficial and SCFA-producing bacteria [105–108].

In terms of single component of TCM, berberine, an isoquinoline alkaloid derived from Coptis chinensis, can successfully restore the diversity and structure of gut microbiota, regulate microbial metabolites, and ameliorate gut microbial imbalance in patients with PMOS [109]. Research indicates that quercetin can systematically lower insulin, blood glucose, cholesterol, and TG levels, modulate pituitary-ovarian axis function, mitigate oxidative stress damage associated with PMOS, manage the metabolic environment of patients, and treat PMOS symptoms [110].

Overall, polyphenols and TCM can improve PMOS through multi-dimensional pathways such as restoring gut microbiota balance, ameliorating metabolic dysfunction and suppressing inflammatory responses, showing comprehensive therapeutic potential. However, it is worth noting that current evidence that natural plant compounds and traditional Chinese medicines for improve PMOS by regulating gut microbiota is still mainly from animal experiments. The key molecular targets, the dependence of therapeutic effects on gut microbiota, and the exact clinical benefits in humans still need to be further clarified. Therefore, such interventions are still at the exploratory and adjunctive therapeutic level, and cannot be used as the first-line and definitive treatment for PMOS. In the future, more in-depth mechanism studies and large-sample, long-term clinical trials are needed to clarify the molecular targets, efficacy and safety of these compounds, so as to promote their clinical transformation and application.

### Glucose-lowering and anti-obesity drugs

PMOS patients with insulin resistance are accompanied by impaired insulin receptor-related signaling pathways, which will be further aggravated by obesity and its related phenotypes such as visceral fat accumulation, chronic inflammation and oxidative stress response. Metformin and GLP-1 receptor agonists can not only induce therapeutic effects via weight reduction but also influence the mechanisms underlying IR, potentially serving as useful treatment alternatives for obese patients with PMOS.

Among these agents, metformin can decrease weight, enhance insulin sensitivity, regulate menstrual cycle and reduce androgen level [111]. In recent years, the regulatory effect of metformin on gut microbiota has attracted increasing attention. Research indicates that metformin can improve metabolic disorders and restore ovulatory function in PMOS rats by activating intestinal AMPK, regulating gut microbiota and increasing the production of beneficial metabolites such as I3A [112]. Clinical studies have confirmed that metformin treatment can effectively reduce body fat rate and visceral fat content in PMOS patients, and improve IR and persistent low-grade inflammation.

Simultaneously, metformin can enhance *Akkermansia muciniphila*, *Clostridium leptum* group, *Faecalibacterium prausnitzii* and other beneficial bacteria, reduce the proportion of harmful bacteria, and rebuild the structure of gut microbiota. Xue et al. [113] discovered that metformin can mitigate PMOS by regulating gut microbiota, decreasing plasma LPS levels, and lowering the concentration of inflammatory mediators, including tumor necrosis factor α, interleukin 6, and interleukin 17A, in individuals with PMOS. Significantly, metformin treatment may normalize altered fecal SCFA levels in patients with PMOS, suggesting that metformin can improve intestinal microenvironment by regulating SCFA metabolism [114]. Nonetheless, metformin is prone to gastrointestinal adverse reactions, which limits the long-term medication compliance of patients.

The weight-loss and metabolic enhancement benefits of GLP-1 receptor agonists provide unique potential for expanding the treatment options of PMOS. Semaglutide can reestablish the movement rhythm of PMOS mice via the PI3K/AKT/mTOR pathway, rectify serum hormone imbalances, enhance glucose tolerance, and mitigate autophagy and ovarian oxidative damage in PMOS animals [115]. Semaglutide can also reduce ovarian tissue inflammation and enhance PMOS phenotype through AMPK/SIRT1/NF-κB signaling pathway [116].

In a clinical study, 3 mg liraglutide was given daily to obese and non-diabetic PMOS women for 32 consecutive days. After short-term treatment, improvements in body weight, hyperandrogenism and menstrual cyclicity were observed [117]. Exenatide combined with metformin has a good effect in reducing body weight, decreasing BMI and waist circumference, and improving blood glucose and insulin sensitivity in overweight or obese PMOS women, with acceptable short-term adverse reactions [118]. Nonetheless, the precise mechanism of GLP-1 receptor agonists in improving metabolic and reproductive abnormalities related to PMOS is still not fully elucidated, and most of the existing studies lack long-term follow-up data. The persistence of weight-loss effect and the long-term metabolic benefit of GLP-1 receptor agonists still need more clinical verification [119].

Orlistat plays an anti-obesity role by inhibiting gastrointestinal lipases and reducing the absorption of dietary fat. Current randomized controlled trial data confirm that orlistat has a certain effect in reducing weight, decreasing HOMA-IR index, insulin level and testosterone concentration in PMOS patients, but the incidence of adverse reactions related to orlistat is high [120].

Metformin, GLP-1 receptor agonists, orlistat and other hypoglycemic and weight-loss drugs have been confirmed to reduce body weight, improve insulin sensitivity and normalize metabolic disorders, so they provide feasible treatment choices for obese PMOS patients.

Although various pharmacological treatments will be accompanied by alterations in gut microbiota composition and function, it remains difficult to distinguish the extent to which these changes directly mediate therapeutic effect, or are the secondary consequences of improved metabolism and weight loss. In this context, gut microbiota is more likely to serve a dual role as both a mediator of treatment effects and a responsive biomarker.

## Discussion

### Limitations of current research

Despite the comprehensive synthesis of evidence regarding the associations among gut microbiota, microbial metabolites, and PMOS, this narrative review and the underlying primary literature have several notable limitations that should be acknowledged.

At the review level, although this article adopted a systematic literature search strategy, it remains a narrative review rather than a formal scoping review or systematic review. Therefore, the evidence synthesis may still be influenced by narrative interpretation and selection heterogeneity. To address this concern, detailed information on database sources, search time frame, keyword strategies, inclusion and exclusion criteria, as well as the study selection process has been supplemented in the revised manuscript to enhance methodological rigor and transparency.

At the primary research level, most of the included studies are observational or cross-sectional in design, with a scarcity of large-scale randomized controlled trials and long-term prospective cohort data. Consequently, the causal inference linking gut microbiota dysbiosis to the onset and progression of PMOS remains weak, and most conclusions are restricted to associative relationships rather than definitive causal pathways.

In addition, substantial heterogeneity exists across included studies in terms of microbial detection techniques, with some studies using 16S rRNA gene sequencing and others adopting metagenomic or metabolomic approaches. Study populations differ significantly in ethnicity, obesity status, age, and PMOS phenotypic classification, and the application of Rotterdam criteria also varies across research centers. These inconsistencies contribute to divergent microbial signatures and metabolic profiles, limiting cross-study comparability and the generalizability of conclusions.

Mechanistically, most studies focus on correlative analysis between microbial alterations and clinical phenotypes, whereas detailed and validated signaling pathways through which key microbial metabolites regulate insulin sensitivity, androgen synthesis, inflammatory responses, and ovarian function remain insufficiently clarified. The functional validation of specific bacterial genera and critical metabolites is mostly derived from animal models, lacking direct and reliable evidence in humans.

Finally, most microbiota-targeted interventions, including probiotics, prebiotics, fecal microbiota transplantation, natural compounds, and related pharmacological agents, are supported by preclinical experiments or small-scale preliminary clinical trials. Issues such as optimal strain selection, therapeutic dosage, intervention duration, clinical reproducibility, long-term efficacy, and safety profiles have not been systematically elucidated, hindering the translation of microbiota-based strategies into routine clinical practice for PMOS.

### Conclusion and future perspectives

In summary, accumulating evidence indicates that gut microbiota and PMOS are linked through complex and tightly interconnected interactions, with gut microbial dysbiosis emerging as a critical regulatory node bridging metabolic abnormalities, endocrine dysfunction, and ovarian impairment. During the onset and progression of PMOS, alterations in gut microbiota may contribute to the development of core phenotypes, including insulin resistance, hyperandrogenism, and obesity, by modulating insulin sensitivity, chronic low-grade inflammation, and androgen homeostasis. An increasing body of evidence further suggests that microbiota-derived metabolites act as key mediators in gut microbiota-host interactions. Through the regulation of metabolic pathways, immune responses, and endocrine signaling networks, these metabolites collectively drive the pathophysiological changes associated with PMOS. Microbiota-targeted interventions have shown promising potential in improving metabolic and endocrine outcomes; however, their underlying mechanisms, causal relevance, and clinical benefits remain to be fully elucidated.

This narrative review was performed using a systematic and transparent literature search strategy with well-defined database sources, search terms, time frame, and rigorous inclusion and exclusion criteria, which greatly improved the methodological reliability and quality of evidence synthesis. Future research should therefore prioritize well-designed studies with clearly defined disease phenotypes, integrating longitudinal follow-up, causal inference approaches, and multi-omics analyses to systematically delineate the key regulatory pathways through which gut microbiota and their metabolites contribute to PMOS. As understanding of the gut–metabolism–endocrine–ovary axis continues to advance, precision intervention strategies based on individual gut microbiota characteristics may offer new theoretical foundations and therapeutic directions for the long-term management of PMOS and the improvement of overall patient health outcomes.

## Plain language summary

Polyendocrine metabolic ovarian syndrome (PMOS), is a common condition affecting many women of reproductive age and is often accompanied by hormonal imbalance, metabolic disorders, and fertility challenges. In recent years, increasing research has suggested that the gut microbiota—the community of microorganisms in the digestive tract—may play an important role in the development of PMOS.

This review summarizes current findings on the relationship between gut microbiota and PMOS and explores how changes in gut microbes may affect metabolism, hormone regulation, and ovarian function. We propose a unified biological pathway called the “gut–metabolism–endocrine–ovary axis” to explain how the gut interacts with multiple systems to influence PMOS.

Understanding these interactions may improve our knowledge of PMOS and support the development of new prevention and treatment strategies.
